# Carbon and energy intensity of the USA and Germany. A LMDI decomposition approach and decoupling analysis

**DOI:** 10.1007/s11356-022-22978-x

**Published:** 2022-09-15

**Authors:** Eleni Koilakou, Emmanouil Hatzigeorgiou, Kostas Bithas

**Affiliations:** 1 Institute of Urban Environment & Human Resources, Department of Economic & Regional Development, Panteion University, 29 Aristotelous Street, GR-17671, Kallithea, Athens, Greece; 2 Energy Management Laboratory, Department of Environment, University of the Aegean, University Hill, 81100 Lesvos, Greece

**Keywords:** LMDI decomposition analysis, CO_2_ emissions, Energy intensity, Decoupling effect, Scenario analysis, USA, Germany

## Abstract

The present study investigates the driving factors leading energy and carbon intensity of the economies of the USA and Germany, being two economies with different structures and dependencies on energy while they are among the world’s economic and geopolitical leading players. Both decoupling and decomposition analysis (overall and sectoral) are applied to identify and rank factors defining carbon and energy intensity during the period from 2000 to 2017, with the so-called financial crisis being within this period. The decoupling analysis denotes that these advanced economies have reached a weak decoupling status, while decomposition analysis confirms the leading role of energy intensity in CO_2_ emissions, followed by the income (positive contribution) and the energy mix emerge as important factors with population (positive contribution) trend to be an additional factor only in the USA.

**Supplementary Information:**

The online version contains supplementary material available at 10.1007/s11356-022-22978-x.

## Introduction

Trends of CO_2_ emissions as well as of carbon intensity of the economy are of prominent importance of contemporary economic and energy policies. CO_2_ emissions and carbon intensity are the outcome of several factors including the efficiency by which the energy is being used, the energy mix (fossil fuels versus renewables), the growth trends, and structure of the economy as well as income and population size and trends. The evolution and ranking of the driving forces of CO_2_ emissions is an issue of scientific and policy importance. The present study attempts an investigation over the carbon and energy intensity of the great economies of the USA and Germany attempting to identify and evaluate the key driving forces.

The international literature proves that the driving forces of economic and demographic growth, geopolitical changes, international trade, and western society’s lifestyle are inducing high energy consumption and coupling between growth and CO_2_ emissions (Hatzigeorgiou et al. 2008). Various methods have been developed to evaluate and rank the factors driving energy consumption and CO_2_ emissions, including the index decomposition analysis (IDA) (Ang 1999; Wang et al. 2018) and decoupling analysis (Tapio 2005). The present article investigates the driving forces behind the trends of the energy-related CO_2_ emissions in Germany and in the USA from 2000 to 2017. The paper attempts a decomposition analysis by means of the logarithmic mean divisia index (LMDI) method while combining it with a decoupling analysis estimating a broad set of decoupling indexes. The leading role of these two advanced economies in the continents of America and Europe as well as at the global level, their different structures and evolutionary trajectories, their different mixtures of energy, and climate policies make the comparison between these two giant economies of significant importance.

For the USA, a number of studies investigate the driving factors of CO_2_ emissions. Vinuya et al. (2010) analyzed the trends of CO_2_ emissions for the US economy employing the LMDI technique. The authors concluded that the decline of energy intensity was the driving factor for the reduction of CO_2_ emissions. Baldwin and Wing (2013) studied the spatiotemporal evolution of CO_2_ emissions for the US economy employing a permutation of Kaya identity, over the period 1963–2008. The results denoted that energy efficiency improvement and compositional shifts were the causal factors for the decrease of CO_2_ emissions. Shahiduzzaman and Layton (2017) analyzed the feasibility of the greenhouse gas (GHG) target for 2025, employing the additive LMDI technique in aggregate and sectoral level. Their aggregate analysis indicated that gross domestic product (GDP) and population effects were the main contributors on the increase of total CO_2_ emissions for the years 2000–2014, while energy intensity had the biggest negative contribution. Moreover, their sectoral analysis over the 1973–2014 period denoted that energy intensity contributed about 78% of total mitigation in the industrial sector, about 63% in the transport sector, and about 84% in the commercial sector.

Wang et al. (2018) compared CO_2_ emissions in the China and the USA by an LMDI approach combined with a decoupling analysis of CO_2_ emissions from economic growth. The study showed that the main reasons for the decrease of CO_2_ emissions in China and the USA are income and population effects followed by the energy intensity and the energy mix effects. Jiang et al. (2019), based on Cobb-Douglas (C-D) production function and LMDI technique, examine the evolution of CO_2_ emissions in the USA. The study quantified the impact of the technological status on CO_2_ emissions for the USA. Song et al. (2019) established a two-dimensional model for the relationship between economic development and CO_2_ emissions in China and the USA. The methodology was based on Tapio (2005) decoupling index and environmental Kuznets curve (EKC) for 1965–2016 time span. The results depicted that strong decoupling has not achieved. Wang et al. (2020) implied an aggregate and sectoral decomposition analysis of CO_2_ emissions for the USA over 1997–2016. The study resulted that the main influencing factor is the scale effect (income and population), while the technology effect (energy intensity and emission coefficient) is the key driving force in mitigating emissions. For the residential, commercial, industrial, and transportation sectors, the energy intensity effect is the main factor slowing the growth of emissions over the study period. In contrast, income effect is the primary driving force behind the increase in emissions over the same period.

Regarding the case of Germany, the studies on the identification of the driving forces of CO_2_ emissions are numerous. Bhattacharyya and Matsumura (2010) analyzed the decline of greenhouse gas emissions in European Union (EU) over the period 1990–2007, employing the LMDI decomposition technique. Their paper revealed that the reduction of the energy intensity in the EU-15 is attributable to significant improvements by large emitters such as Germany and the UK. Gonzalez et al. (2014) suggested environmental and energy strategies in order to control CO_2_ emissions using LMDI technique in the EU-27. The results signified the negative impact of the energy intensity and fuel mix effects and the positive impact of the population, income, and emission factor for the change of the CO_2_ emissions.

Ward et al. (2016) established a simple model based on IPAT (impact = population × affluence × Technology) equation questioning the possibility of decoupling of GDP growth from environmental impacts for selected countries. The model showed that GDP growth cannot be decoupled from energy use. Cohen et al. (2018) evince the existence of decoupling between GHG emissions and economic growth using a simple trend/cycle decomposition for Germany (1990–2014). Sadorsky (2020) studied the driving factors of energy-related CO_2_ emissions, applying the LMDI technique. The decrease in energy intensity was the key driver behind declining CO_2_ emissions, while an increase in GDP was the biggest force enlarging CO_2_ emissions. Haein et al. (2020) used the LMDI technique for decomposing CO_2_ emissions from electricity generation, employing a comparative analysis for 36 OECD countries. The study proved that the improvement in electricity intensity (ELI), calculated as electricity generation per GDP (EL/GDP), and the decrease in the share of thermal generation were the main effects for the reduction of CO_2_ emissions.

Other scholars, in recent papers, focused on the evolution of GHGs for China as a large economy, by means of decomposition or/and decoupling analysis (Fatima et al. 2019; Yang et al. 2020; Zhang et al. 2020; Hu et al. 2021).

The objective of this paper is to evaluate and analyze the energy and CO_2_ intensity of the USA and Germany while they are among the world’s economic and geopolitical leading players, attending an issue with high policy relevance under the current energy status of the global economy: revised climate targets, increasing energy prices, and risks related with energy supply. Remarkably, the GDP of German economy accounted for 24.07% of the EU (The World Bank Group 2020). In 2018, the global share of CO_2_ emissions for USA is 14.7% (IEA 2020a) and the US GDP accounted for 21.54% of the global GDP (The World Bank Group 2020). Although a number of past studies have concentrated on CO_2_ emissions and economic output for various countries, a comparative analysis of driving factors for energy-related CO_2_ emissions, especially between the two major economies of USA and Germany, is not presented in the energy literature. The proposed analysis could provide valuable insight.

First, we conduct a descriptive comparative analysis between the two economies, delineating their energy status and we investigate a “business as usual” scenario, examining whether the recent emission targets of the two economies are attainable under their current underling trends. Next, we are employing a set of decoupling indexes to explore the linkage between the energy-related CO_2_ emissions and economic growth. Finally, we are applying robust LMDI techniques in order to rank and quantify the driving forces leading CO_2_ emissions. A sectoral decomposition analysis is also applied in order to contribute in more specific policy recommendations. The period under investigation is from 2000 to 2017.

Regarding the environmental policies, Germany follows the Climate Action Law (the German first national climate law) which has been included in German government coalition’s 2030 climate package. Its targets can be summarized by the reduction in greenhouse gas emissions up to 55% by 2030 and the greenhouse gas neutral by 2050. Those objectives have been inspired for the respective EU policies (BMWI 2019; European Commission 2020a). On the other hand, the target of the US economy is to reduce CO_2_ emissions by 26–28% in 2025 compared to 2005^1^, as submitted to the UNFCCC (United Nations Framework Convention on Climate Change).

The energy and climate policy targets of the two countries for the study period 1990–2017 are summarized in the Table 1.

**Table 1:** Emission goals for the USA and Germany (1990–2017)

***Milestone regulations (1990–2017)***						
Germany	1990: Federal government committed to reduce carbon dioxide emissions to 25% below 1990 levels by 2005. (First Initiative 1990) Also, Germany agreed that by 2020, GHG emissions would drop 40% from 1990 levels (Bailey and Rupp 2004).	1999: Germany signed the Kyoto Protocol and has committed to reduce its emissions to 21% below 1990 levels between 2008 and 2012 (Telli et al. 2020).	2007: The parliament of the EU approved the following goals for the year 2020^a,b^:	2010: The updated government targets are to reduce total emissions by at least 40% by 2020 and at least 55% by 2030, compared with 1990 levels (2010 Energy Concept) (IEA 2020b).	2011: German government announced the Energiewende (energy transformation) and decided to reduce the amount of fossil fuels from 80% of energy supply to 20% by 2050 (Lu et al. 2020).	2016: Germany follows the European Council agreement submitted to UNFCCC for a reduction in GHG within the EU of at least 40% by 2030, compared with 1990 (BMUB 2016).
			-20% cut in greenhouse gas emissions (from 1990 levels)			
			-20% of EU energy from renewables			
			-20% improvement in energy efficiency.			
The USA	1990: Under the UNFCCC, the USA committed to the voluntary goal of holding greenhouse gas emissions at the end of the 1990s decade to their 1990 levels (Blodgett et al. 2002).	1997: In the 1997 Kyoto Protocol to the UNFCCC, the USA participated in negotiations that ended with agreement on further reductions that could become legally binding (Blodgett et al. 2002).	2002: The Administration rejected the Kyoto Protocol. The goal is to reduce GHG intensity by 18% over the next 10 years through voluntary activities, which means that the 183 metric tons of carbon emissions (MMTCE) per million dollars of GDP that the United States is emitting in 2002 would fall to 151 MMTCE per million dollars of GDP in 2012 (Blodgett et al. 2002).	2014: USA’s target as submitted to the UNFCCC, aims at reducing CO_2_ emissions by 26–28% in 2025 compared to 2005^c^.		2017: Under the Paris Agreement, the USA promised to achieve a reduction of about 25% by 2025 compared with 2005 levels(2016)^d,e^.
						The next year, it announced the USA withdrawal from the Paris Agreement^f^. In 2021, the USA rejoined the Paris Agreement^g^.

^a^https://www4.unfccc.int/sites/submissions/indc/Submission%20Pages/submissions.aspx

^b^https://ec.europa.eu/clima/policies/strategies/2020_en

^c^https://www.eea.europa.eu/themes/climate/trends-and-projections-in-europe/trends-and-projections-in-europe-2017/overall-progress-towards-the-european

^d^https://unfccc.int/node/61231

^e^https://unfccc.int/process-and-meetings/the-paris-agreement/the-paris-agreement

^f^https://www.state.gov/on-the-u-s-withdrawal-from-the-paris-agreement/

^g^https://www.state.gov/the-united-states-officially-rejoins-the-paris-agreement/

Table 1 reveals that the policy regulations for Germany usually set a strict target in order to achieve a significant reduction of the CO_2_ emissions in the future. On the other hand, the US energy and environmental policy set a more achievable target for the reduction of CO_2_ emissions.

These two economies are among the largest carbon-emitting countries in the world (15.7% of global CO_2_ emissions in 2020) as stated in recent COP26. Consequently, their environmental and energy status is of great interest in the context set by the goals of the Paris Agreement.

The remainder of the paper is organized as follows. The second and third sections present the methodology used and the data analysis, respectively. Results and discussion are described in the fourth section 4. The fifth section summarizes the conclusions.

## Methods

We conduct a decoupling analysis between energy-related CO_2_ emissions (*C*) and economic growth for the USA and Germany. Decoupling CO_2_ emissions with economic development is essential for environmental management.

We estimate the decoupling index (*DI*) for the standard *C/GDP* ratio:

1 $${\mathrm{DI}}_{\mathrm{GDP}}=\frac{\Delta(C)}{\Delta\left(\mathrm{GDP}\right)}=\frac{\left(C_t-C_{t-1}\right)/C_{t-1}}{\left({\mathrm{GDP}}_t-{\mathrm{GDP}}_{t-1}\right)/{\mathrm{GDP}}_{t-1}}$$

and we estimate the DI for the *C*/income ratio:

2 $${\mathrm{DI}}_{\mathrm{Inc}}=\frac{\Delta(C)}{\Delta\left(\mathrm{income}\right)}=\frac{\left(C_t-\;C_{t-1}\right)/C_{t-1}}{\left({\mathrm{income}}_t-{\mathrm{income}}_{t-1}\right)/{\mathrm{income}}_{t-1}}$$

where national income as

3 $$\mathrm{Inc}=\frac{GDP}{P}$$

We also set the EI_GDP_ *= E*/GDP and EI_Inc_ *= E*/income energy intensity ratios. The CI_Inc_ *= C*/income carbon intensity ratio is proposed as an indicator that approximates better than the CI_GDP_ *= C*/GDP ratio the real-world properties of production. In accordance with the existing literature, the indexes DI_Inc_ and CI_Inc_ denote weaker decoupling trends than the DI_GDP_ and CI_GDP_, respectively (Bithas and Kalimeris 2013; Bithas and Kalimeris 2018). The categorization of the decoupling states is based on the study of Tapio (2005).

Time series decomposition analysis is employed to assess the evolution of the decomposition factors during the 2000–2017, in the USA and Germany. LMDI technique (additive and multiplicative) is applied to determine and rank the causal effects leading the trends of CO_2_ emissions in a comparative basis. This technique has emerged as the most preferred IDA technique among researchers and analysts for ease of formulation and simplicity sake. Moreover, LMDI technique is extensively utilized because it results in incomplete decomposition and gives zero residual term (Hatzigeorgiou et al. 2008; Yasmeen et al. 2020).

The following variables are defined for each year:

*i*: fuel type (coal, oil, natural gas, renewable resources)
*E*_*i*_: energy consumption of fuel type *i* (Mtoe)
*E*: total energy consumption (Mtoe)
*C*: total CO_2_ emissions (MtCO_2_)
*C*_*i*_: CO_2_ emissions from fuel type *i* (MtCO_2_)
*Y*: GDP (million 2010US$ for Germany/million 2012US$ for the USA)
*P*: population (in million people)

The energy-related CO_2_ emissions (C) are given by the equation:

4 $$C={\sum}_{i=0}^4I\ {S}_i\ P\mathrm{In}c\ {F}_i={\sum}_{i=0}^4\left(\frac{E}{\mathrm{GDP}}\right)\left(\frac{E_i}{E}\right)P\left(\ \frac{\mathrm{GDP}}{P}\ \right)\left(\ \frac{C_i}{E_i}\ \right)$$

where

- Energy intensity as

5 $$I=\frac{E}{\mathrm{GDP}}$$

- Energy structure as

6 $${S}_i=\frac{E_i}{E}$$

- Emission factor as

7 $${F}_i=\frac{C_i}{E_i}$$

- Population as

8 $$P$$

In additive LMDI technique, the difference in CO_2_ emission levels between 2 years can be expressed in MtCO_2_ as follows:

9 $$\Delta {C}_{\mathrm{tot}}=\Delta {C}_{\mathrm{p}}+\Delta {C}_{\mathrm{inc}}+\Delta {C}_{\mathrm{int}}+\Delta {C}_{\mathrm{f}}+\Delta {C}_{\mathrm{s}}+{C}_{\mathrm{T}}-{C}_0$$

where Δ*C*_tot_ is the change of total CO_2_ emissions.

The relevant formulas for the decomposition factors are as follows:

10 $$\Delta {C}_{\mathrm{p}}={\sum}_{i=1}^4\frac{\left({C}_{i,T}-{C}_{i,0}\right)}{\ln \left({C}_{i,T}/{C}_{i,0}\right)}\ln \left(\frac{P_T}{P_0}\right)$$

11 $$\Delta {C}_{\mathrm{inc}}={\sum}_{i=1}^4\frac{\left({C}_{i,T}-{C}_{i,0}\right)}{\ln \left({C}_{i,T}/{C}_{i,0}\right)}\ln \left(\frac{Inc_T}{Inc_0}\right)$$

12 $$\Delta {C}_{\mathrm{int}}={\sum}_{i=1}^4\frac{\left({C}_{i,T}-{C}_{i,0}\right)}{\ln \left({C}_{i,T}/{C}_{i,0}\right)}\ln \left(\frac{I_T}{I_0}\right)$$

13 $$\Delta {C}_{\mathrm{f}}={\sum}_{i=1}^4\frac{\left({C}_{i,T}-{C}_{i,0}\right)}{\ln \left({C}_{i,T}/{C}_{i,0}\right)}\ln \left(\frac{F_{i,T}}{F_{i,0}}\right)$$

14 $$\Delta {C}_{\mathrm{s}}={\sum}_{i=1}^4\frac{\left({C}_{i,T}-{C}_{i,0}\right)}{\ln \left({C}_{i,T}/{C}_{i,0}\right)}\ln \left(\frac{S_{i,T}}{S_{i,0}}\right)$$

where Δ*C*_p_ is the change of population, Δ*C*_inc_ the change of income, Δ*C*_int_ the change of the energy intensity, Δ*C*_f_ the change of the emission factor, and Δ*C*_s_ the change of the energy structure.

For the multiplicative LMDI technique, the formulas are demonstrated in Appendix A.

Sectoral decomposition analysis is also implied for the US and German economies. Period-wise multiplicative LMDI technique is applied, where 0 and *T* stands for the benchmark year (2000) and the final year (2017). The economy sectors are classified as industrial, commercial, residential, and transportation (where *i* represents the type of sector), while the energy-related CO_2_ emissions (C) are described as follows:

15 $$C={\sum}_{i=0}^4I\ \mathrm{GDP}\ {F}_i$$

where

- Energy intensity as

16 $$I=\frac{E}{\mathrm{GDP}}$$

- Emission factor as

17 $${F}_i=\frac{C_i}{E_i}$$

- Gross domestic product as

18 $$GDP$$

The change scheme in sectoral CO_2_ emission levels between 2 years can be expressed as follows:

19 $${D}_{tot}={D}_{\mathrm{gdp}}{D}_{\mathrm{int}}{D}_{\mathrm{f}}={C}_{\mathrm{T}}/{C}_0$$

where *D*_tot_ is the change of energy-related CO_2_ emissions caused by GDP effect (*D*_gdp_), energy intensity effect (*D*_int_), and sectoral emission factor effect (*D*_f_).

All the indexed ratios in figures are calculated to a base year (base year = *t*_0_ =100):

20 $${\mathrm{Indexed}\;V\mathrm{alue}}_{t1}=100+\frac{\left({Value}_{t1}-{Value}_{t0}\right)}{Value_{t0}}$$

## Data and analysis

### Data comparison for the economies of the USA and Germany

The trends of the key variables of our study are presented in Fig. S1–S6 in the supplementary information file. Figures S1 and S2 indicate the trends of the main fuels in energy consumption: oil, natural gas, renewable resources, and coal, with data drown from the International Energy Agency (IEA 2020a). Nuclear energy may not be listed separately in the final energy consumption but is used for the aggregate calculation of renewable resources, since the nuclear energy has neutral C0_2_ emissions. The energy consumption of nuclear energy is estimated as a sub-category of renewable resources for Germany and the USA (IEA 2020a). Electricity is not considered to be a separate fuel; it is taken into account via the original fossil fuels that are utilized to produce electricity (Hatzigeorgiou et al. 2010). Renewable resources for Germany present a remarkable increase by 220% through the last 17 years, while in the USA the increase of renewables is approximately 52%. The share of oil, the fuel with the largest energy consumption, declines for both Germany (− 17.5%) and the USA (− 6.7%).

To estimate CO_2_ emissions from primary fuel consumption, we adopted the OECD emission coefficients (IPCC 2006) depicted in Table S1 in the supplementary information file. Energy-related CO_2_ emissions follow significant fluctuations (Fig. S3), with remarkable decrease for the 2006–2007 time period in Germany and 2007–2009 in the USA. Remarkably, these time periods coincide with the onset of the financial crisis.

The World Bank Open Data are adopted for the aggregate values of Germany’s GDP (The World Bank Group 2020), while the GDP of the USA is based on data from Bureau of Economic Analysis (BEA 2020). An upward trend of GDP for both countries is shown in Fig. S4, with the exception of the 2007–2009 time period; GDP for Germany declined by 5% while for the US economy declined by 8%.

All data for population (Fig. S5) are retrieved from World Bank Open Data^2^. The population in the USA increases linearly, while Germany’s population remains approximately constant, with a slightly decrease from 2009 to 2011. An upward trend in income is observed in both countries, similar to GDP (Fig. S6).

Summarizing all the above-mentioned facts, Table 2 depicts the physiology of the two economies and the analogy of their key variables for the years 2000 (a) and 2017 (b).

**Table 2:** The physiology of the USA and German economies for the years 2000 (a) and 2017 (b)

*a.* *Key variables - 2000*	*USA*	*Germany*	*USA/Germany*
Energy consumption (*Mtoe*)	1546.3	231.4	6.7
CO_2_ emissions (*MtCO*_*2*_)	3411.8	515.5	6.6
GDP (*million 2010 US$)*	12,620,268.4	3,118,322.5	4.0
Population (*million people*)	282.2	82.2	3.4
Income (*2010 US$*)	44,721.0	37,935.8	1.2
*b.* *Key variables* - *2017*	*USA*	*Germany*	*USA/Germany*
Energy consumption(*Mtoe*)	1520.5	227.0	6.7
CO_2_ emissions (*MtCO*_*2*_)	3163.3	447.6	7.1
GDP (*million 2010 US$)*	17,348,626.6	3,878,004.0	4.5
Population (*million people*)	325.1	82.7	3.9
Income (*2010 US$*)	53,364.0	46,892.4	1.1

Although the aggregate energy consumption for the two economies remains stable (USA/Germany = 6.7), the respective analogy for CO_2_ emissions increased from 6.6 to 7.1 (2000–2017). This reflects that German policy measures for decarbonization of the economy are more effective than those of the USA.

In our attempt to test the efficiency of the existing environmental policies for both economies, we set a “business as usual” scenario. For the case of Germany, we attempt to forecast the evolution of CO_2_ emissions from 2018 to 2030. The years 1990 and 2030 are the landmarks of the German climate policy. By using historical data for the years 2000–2017, we estimate the average annual rate of CO_2_ emissions (− 0.75%) according to the following formula:

21 $$\mathrm{Average}\;\mathrm{annual}\;\mathrm{rate}\;\mathrm{of}\;{\mathrm{CO}}_2\;\mathrm{emissions}=\frac{\sum_1^N\left(\mathrm{annual}\;\%\;\mathrm{change}\;\mathrm{of}\;{\mathrm{CO}}_2\;\mathrm{emissions}\right)}{N\;}$$

where *N* is the number of years.

We assume this rate will persist for the years 2018–2030, implying a linear projection of the current average rate of CO_2_ emission reduction in Germany. According this way, we also forecast the evolution of CO_2_ emissions from 2018 to 2025 for the case of the USA. The average annual rate of change of CO_2_ emissions for the years 2018–2025 is equal to – 0.41%, as defined by using historical data for the years 2000–2017. The forecast results imply that CO_2_ emission targets cannot be achieved under the “business as usual” scenario for both economies: the German economy remains apart by 52.8% from the 2030 optimistic target, while the USA by 18.7% from the 2025 emission levels.

### Data for sectoral analysis in the USA and Germany

In this subsection we perform a descriptive sectoral analysis for the USA and Germany economy. The following sectors are selected according to the EIA (US Energy Information Administration 2020a) approach:

- Residential sector: Utilities—household consumption
- Commercial sector: Private services (financial, business, educational, health and food services) and government services
- Industrial sector: Agriculture, mining, construction, and manufacturing
- Transportation sector: Transportation and warehousing

**Fig. 1:**
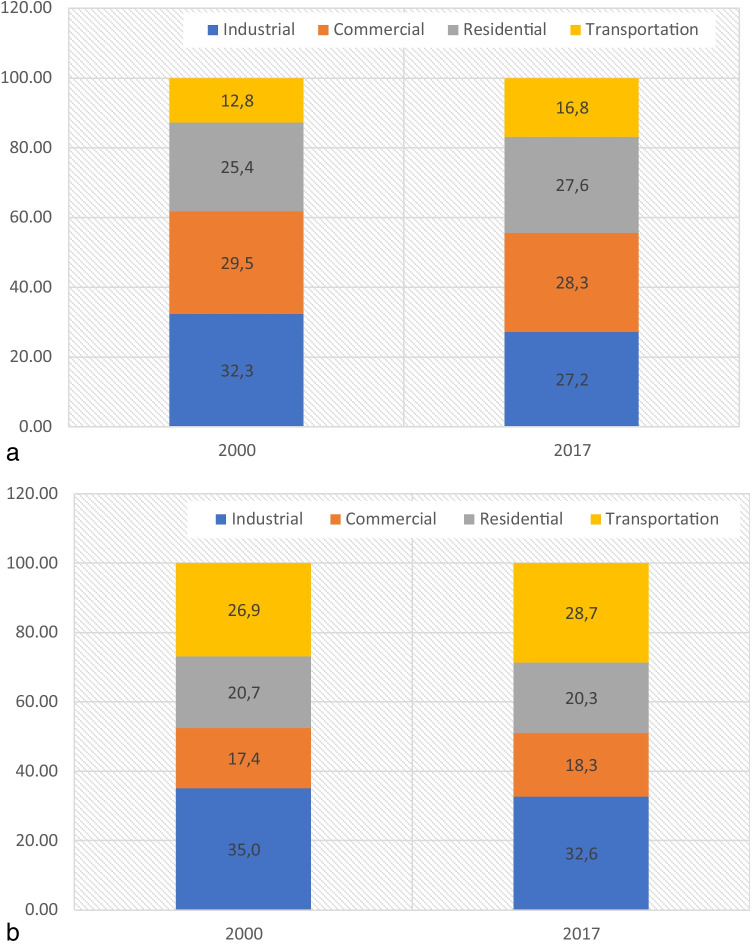
**a** Percentage (%) of energy consumption for the sectors of the German economy (2000 and 2017). **b** Percentage (%) of energy consumption for the sectors of the US economy (2000 and 2017)

Figure 1 presents the energy consumption per sector for Germany (1a) and the USA (1b). The data are retrieved from IEA (IEA 2020a).

In 2000, the industrial sector is responsible for 32.3% of the energy use of the German economy. After 17 years, the industrial sector is responsible for 27.2% of the energy use. For the USA, the share of industrial sector has a reduction of 2.4% (2000–2017).

**Fig. 2:**
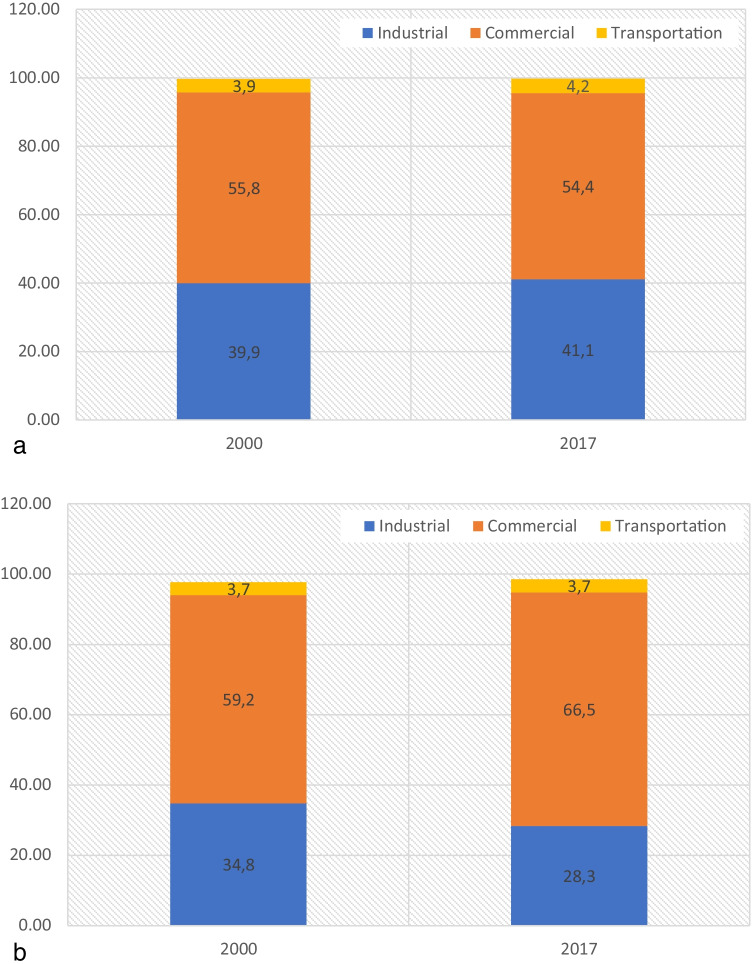
**a** Percentage (%) of GDP for the sectors of the German economy (2000 and 2017). **b** Percentage (%) of GDP for the sectors of the US economy (2000 and 2017)

Figure 2 depicts the GDP per sector for Germany (2a) and the USA (2b). The data are retrieved from Eurostat (European Commission 2020b) and BEA (2020), respectively.

Figure 2a denotes that the structure of the German economy remains almost stable during the study period. Regarding the structure of the US economy, the commercial sector contributes the most value added in 2000 and 2017, while the GDP share for industrial sector is down over 6.5% from 2000 to 2017. These changes demonstrate the USA’s “turning point” as a service-oriented economy in accordance with the relevant literature (Yao et al. 2015; van Neuss 2019). The share of residential sector is negligible for the two economies.

**Fig. 3:**
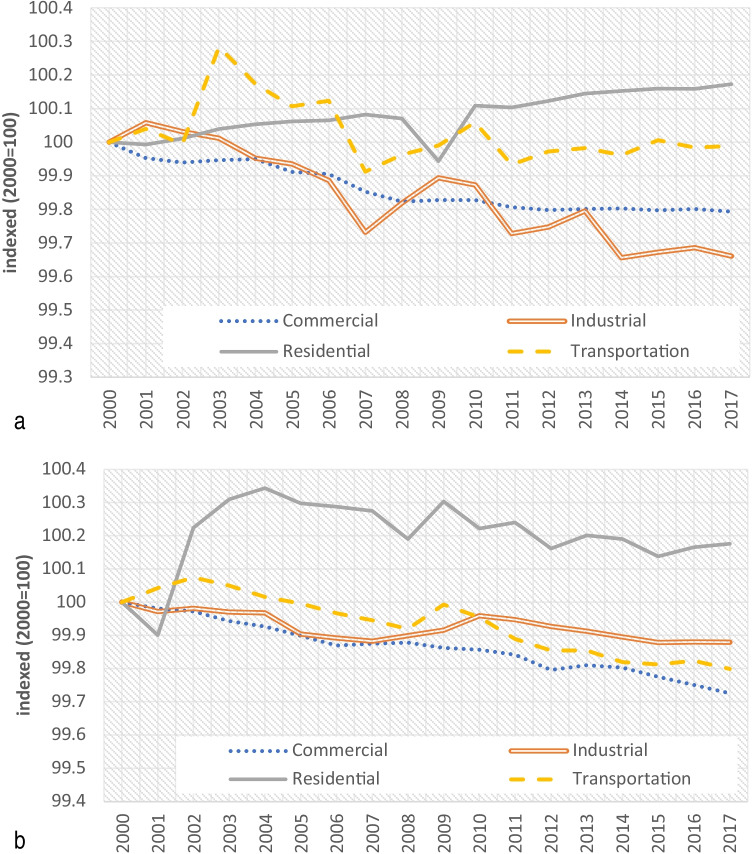
**a** Energy intensity per sector in Germany from 2000 to 2017 (indexed 2000 = 100). **b** Energy intensity per sector in the USA from 2000 to 2017 (indexed 2000 = 100)

Figure 3a presents the energy intensity per sector for the German economy.

As can be seen, energy intensity trends for commercial sector have been gradually decreasing since 2000. The energy intensity for the industrial sector presents fluctuations with a total decreasing trend − 34% in the period 2000–2017.

Figure 3b shows the energy intensity per sector for the US economy.

Decreases in the energy intensity for transportation and industrial sectors denote energy efficiency improvements and structural changes in the economy (Tol et al. 2009; Herrendorf et al. 2014; van Neuss 2019). Significant reduction in energy intensity of commercial sector (− 28%) induces possible rebound effect.

## Results and discussion

### Decoupling analysis of energy-related CO_2_ emissions from economic growth

In this subsection we attempt to interpret the link between economic growth and energy-related CO_2_ emissions by means of a decoupling analysis.

**Fig. 4:**
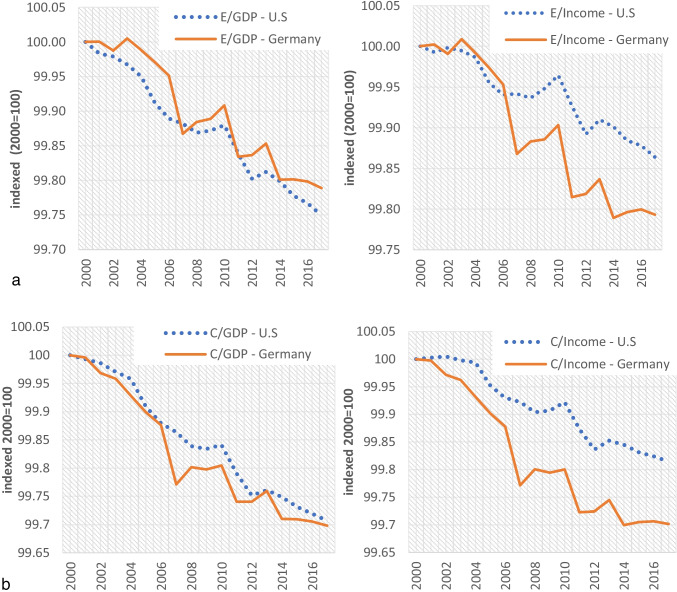
**a** Energy intensity indexed for the USA and Germany from 2000 to 2017 (indexed 2000 = 100). **b** Carbon intensity indexes for the USA and Germany from 2000 to 2017

EI as the key index of the link between economy and energy consumption is often used to assess the energy efficiency of a particular economy, indicating how well the economy “converts” energy into monetary output (Martínez et al. 2019). Figure 4a depicts the trends of energy intensity indexes: EI_GDP_ and EI_Inc_. The declining trends of EI indexes demonstrate the so-called decoupling effect, denoting a gradual delinkage of growth from energy use. The EI_GDP_ index defines similar decreasing trend for both countries, while the EI_Inc_ index denotes a weaker decoupling effect in the USA than Germany.

Figure 4b shows the trends of the carbon intensity indexes: CI_GDP_ and CI_Inc_, for the economies of the USA and Germany.

The declining trends of CI_Inc_ index follow similar evolution trajectories with the EI_Inc_ index, denoting also a weaker decoupling effect between CO_2_ emissions and growth in the USA than Germany.

Tables 3a and 3b present estimates of the EI_GDP_, EI_Inc_, CI_GDP_, and CI_Inc_ indexes for Germany and the USA, respectively, as well as their percentage change from 2000 to 2017.

**Table 3:** (a) Carbon intensity and energy intensity comparison for the economy of Germany. (b) Carbon intensity and energy intensity comparison for the economy of the USA

a				
Year	*EI*_*GDP*_	*EI*_*Inc*_	*CI*_*GDP*_	*CI*_*Inc*_
2000	0.0742	0.0061	0.000165	0.013592
2017	0.0585	0.0048	0.000115	0.009540
*2000–2017 (%)*	*− 21.16%*	*− 21.31%*	*− 30.19%*	*− 29.81%*
b				
Year	*EI*_*GDP*_	*EI*_*Inc*_	*CI*_*GDP*_	*CI*_*Inc*_
2000	0.1225	0.0174	0.000136	0.038468
2017	0.0876	0.0151	0.000096	0.031327
*2000–2017 (%)*	*− 28.49%*	*− 13.22%*	*− 29.33%*	*− 18.56%*

Table 3a indicates an important difference between all carbon intensity and energy intensity indexes; the percentage changes of CI_GDP_ and CI_Inc_ are higher than the respective of the EI_GDP_ and EI_Inc_. This denotes that Germany is less carbon-intensive economy than energy intensive.

For the USA, according to Table 3b, the CI_GDP_ and EI_GDP_ indexes follow similar percentage changes through the period 2000–2017, in contrast with the income-based indexes which betray a more intensive decline of CI_Inc_ compared to EI_Inc_.

Next, in order to evaluate the impacts of the financial crisis on the link between economy and energy/emission, the estimates of the decoupling indexes have been done for the following periods:

- the period before the Great Recession (2000–2007)
- the period of the Great Recession (2007–2013)
- the period after the Great Recession (2013-2017)

The analysis follows categorizations proposed by various studies (Wu et al. 2005; Wang et al. 2005; Watson 2014; Bachtrögler 2016). The results are presented by Tables 4a and 4b for the Germany and the USA, respectively.

**Table 4:** (a) Decoupling analysis of energy-related CO_2_ emissions for 2000–2017 in Germany. (b) Decoupling analysis of energy-related CO_2_ emissions for 2000–2017 in the USA

a						
Time period	DI_GDP_	CI_GDP_ (%)	Decoupling state	DI_Inc_	CI_Inc_ (%)	Decoupling state
2000–2007	− 1.56	− 22.89%	*Strong decoupling*	− 1.57	− 22.84%	*Strong decoupling*
2007–2013	0.60	− 1.52%	*Weak decoupling*	0.39	− 3.46%	*Weak decoupling*
2013–2017	0.02	− 8.07%	*Weak decoupling*	0.02	− 5.78%	*Weak decoupling*
b						
Time period	DI_GDP_	CI_GDP_ (%)	Decoupling state	DI_Inc_	CI_Inc_ (%)	Decoupling state
2000–2007	− 0.01	− 13.59%	*Strong decoupling*	− 0.02	− 7.75%	*Strong decoupling*
2007–2013	− 3.04	− 11.95%	*Strong decoupling*	5.18	− 7.61%	*Expansive decoupling*
2013–2017	0.23	− 7.12%	*Weak decoupling*	0.33	− 4.45%	*Weak decoupling*

Before the financial crisis, we observe a strong decoupling state for both economies. For the case of Germany, the shift from strong to weak decoupling state happens in the years of the financial crisis (DI_GDP_ = 0.6 and DI_Inc_ = 0.39, 2007–2013) and remains in that state (DI_GDP_ = 0.02 and DI_Inc_ = 0.02, 2013–2017), while the USA turns into weak decoupling state after 2013 (DI_GDP_ = 0.23 and DI_Inc_ = 0.33, 2013–2017). The financial crisis in the USA (2007–2013) results in negative CO_2_ emission annual growth rates at − 6% and − 4% in 2009 and 2012, respectively. These were the more intensive negative rates from 2000 to 2017, indicating that CO_2_ emissions declined in line with economic recession (US Energy Information Administration 2020b). Τhe same holds for Germany with 2009 and 2011 presented the most intensive reduction of emissions by − 6.2% and − 4.4%, respectively.

### Overall DA of energy-related CO_2_ emissions in Germany and the USA

#### Results of additive LMDI decomposition analysis

We apply a set of LMDI decomposition techniques to investigate the evolution of energy-related CO_2_ emissions, the dependent variable of the study. With this approach we can quantify the effect of each “decomposition” factor on the evolution of CO_2_ emissions.

The results of the decomposition analysis with additive LMDI technique are depicted in Tables 5a and 5b for Germany and the USA, respectively.

**Table 5:** (a) Factor decomposition analysis (additive LMDI) for Germany, 2000–2017 (unit: MtCO_2_). (b) Factor decomposition analysis (additive LMDI) for the USA, 2000–2017 (unit: MtCO_2_)

a					
Year	*ΔC*_*inc*_	*ΔC*_*pop*_	*ΔC*_*str*_	*ΔC*_*int*_	*ΔC*_*tot*_
2000–2001	7.86	0.87	− 2.36	0.22	6.59
2001–2002	− 1.89	0.86	− 7.73	− 6.65	− 15.40
2002–2003	− 3.87	0.28	− 13.94	8.69	− 8.85
2003–2004	5.94	− 0.11	− 7.59	− 8.17	− 9.93
2004–2005	3.74	− 0.27	− 6.78	− 8.69	− 12.01
2005–2006	18.46	− 0.54	− 2.64	− 9.60	5.68
2006–2007	14.10	− 0.61	− 16.19	− 42.40	− 45.11
2007–2008	5.13	− 0.85	8.70	8.68	21.66
2008–2009	− 24.91	− 1.12	− 4.69	2.38	− 28.34
2009–2010	18.71	− 0.68	− 5.49	9.37	21.92
2010–2011	25.19	− 8.19	0.76	− 37.53	− 19.77
2011–2012	1.00	0.81	− 1.24	1.29	1.86
2012–2013	0.68	1.20	2.62	8.54	13.03
2013–2014	7.80	1.82	− 1.83	− 27.45	− 19.67
2014–2015	3.70	3.73	− 0.42	0.16	7.17
2015–2016	6.12	3.53	− 0.86	− 1.75	7.04
2016–2017	9.16	1.66	0.64	− 5.33	6.13
**2000–2017**	**96.93**	**2.40**	− **59.06**	− **108.25**	− **67.98**
b					
Year	*ΔC*_*inc*_	*ΔC*_*pop*_	*ΔC*_*str*_	*ΔC*_*int*_	*ΔC*_*tot*_
2000–2001	− 34.59	33.64	33.69	− 57.53	− 24.79
2001–2002	− 9.37	31.41	− 10.28	− 14.05	− 2.28
2002–2003	47.10	29.19	− 11.74	− 41.14	23.42
2003–2004	91.18	31.89	14.12	− 60.14	77.05
2004–2005	102.00	31.90	− 37.27	− 144.17	− 47.54
2005–2006	42.52	32.97	− 27.77	− 85.07	− 37.35
2006–2007	33.60	32.37	− 31.44	− 28.23	6.29
2007–2008	− 76.08	31.53	− 49.09	− 49.94	− 143.58
2008–2009	− 208.04	27.72	− 29.31	10.57	− 199.06
2009–2010	74.53	25.93	− 1.47	28.26	127.25
2010–2011	44.78	22.71	− 44.04	− 148.98	− 125.52
2011–2012	56.38	22.21	− 16.93	− 136.29	− 74.63
2012–2013	41.90	21.08	− 1.86	38.80	99.93
2013–2014	70.07	22.85	1.92	− 52.32	42.50
2014–2015	52.33	23.11	8.49	− 82.79	1.15
2015–2016	33.92	22.70	− 5.40	− 44.70	6.53
2016–2017	58.61	20.19	15.70	− 72.30	22.20
**2000–2017**	**420.84**	**463.40**	− **192.68**	− **940.01**	− **248.44**

The Bold entries present the total contribution (positive or negative) of the contributing factor during the study period

As shown in Table 5a, in Germany, the income effect was the primary factor contributing to the increase of CO_2_ emissions during 2000–2017, with the exception of the 2008–2009 time period. The CO_2_ emissions cumulatively increased by 96.93 MtCO_2_ because of income growth. The effects of energy intensity (− 108.25 MtCO_2_) and energy structure (− 59.06 MtCO_2_) are the critical factors that induce the decrease of emissions during 2000–2017. This is the result of the federal government act (2014) which adopted measures for the increase of renewable shares in power generation, modernization of fossil fuel power plants, and the development of more co-generation plants (IEA 2020b). The share of renewables presents 3.2 times increase from 2000 to 2017 (Fig. S1), contributed to the mitigation of CO_2_ emissions. The impact of population effect on CO_2_ emissions (2.4 MtCO_2_) is positive but weak.

Table 5b presents the results of the time series LMDI technique for the USA. The first influencing factor to rise emissions is the population effect. This factor eventually contributed to an increase in emissions by 463.40 MtCO_2_ from 2000 to 2017. The income effect exhibits substantial variations during the examined period, leading to an overall positive contribution resulting in an increase of energy-related CO_2_ emissions by 420.84 MtCO_2_ (2000–2017). The USA has a mean annual increase in income about 0.8%. The energy intensity effect was the most critical factor decreasing CO_2_ emissions. The cumulative effect of energy intensity is near to − 940 Mt CO_2_, while the total change of emissions is – 248.44 MtCO_2_. Moreover, changes in the energy structure played a significant role in decreasing CO_2_ emissions (− 192.68 MtCO_2_, 2000–2017). The energy mix marked an importance swift from coal (− 48%) to renewables (+ 52%) from 2000 to 2017.

#### Comparative evaluation of results

**Fig. 5:**
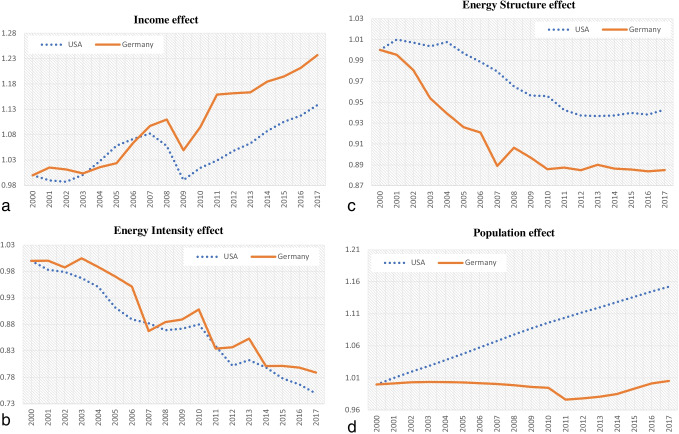
(**a**) Change of energy-related CO_2_ emissions due to the income effect (multiplicative LMDI). (**b**) Change of energy-related CO_2_ emissions due to the energy intensity effect (multiplicative LMDI). (**c**) Change of energy-related CO_2_ emissions due to the energy structure effect (multiplicative LMDI). (**d**) Change of energy-related CO_2_ emissions due to the population effect (multiplicative LMDI)

In this sub-section we analyze the underlying driving forces responsible for the decrease of energy-related CO_2_ emissions, on a comparative basis. We apply the multiplicative LMDI technique to explore and rank contributions of the decomposition factors (Figs. 5a–5d).

The most crucial findings are the following:

1. Total CO_2_ emissions reduced by 13.8% in Germany and by 7.3% in the USA, during 2000–2017. The decreasing trends of energy intensity and the substitution of carbon-intensive coal and oil with renewable resources played positive roles in decreasing CO_2_ emissions. Although it is not clear the reasons behind the trends in energy intensity, they are often attributed to technological advance and a swift of the economy toward services.
2. The energy intensity effect is negative for both economies: the trend for Germany presents a decrease by 21% and by 25% for the USA (Fig. 5b).
3. The energy structure effect influences CO_2_ emissions more in Germany (− 12%) than in the USA (− 6%) (Fig. 5c).
4. The German income effect has greater positive contribution (+ 24%) than the USA (+ 14%) (Fig. 5a).
5. The contribution of population effect, although positive in both economics, is infinitesimal in Germany (+ 0.5%) while strong in the USA (+ 15%) (Fig. 5d).

### Sectoral DA of energy-related CO_2_ emissions in Germany and the USA

**Fig. 6:**
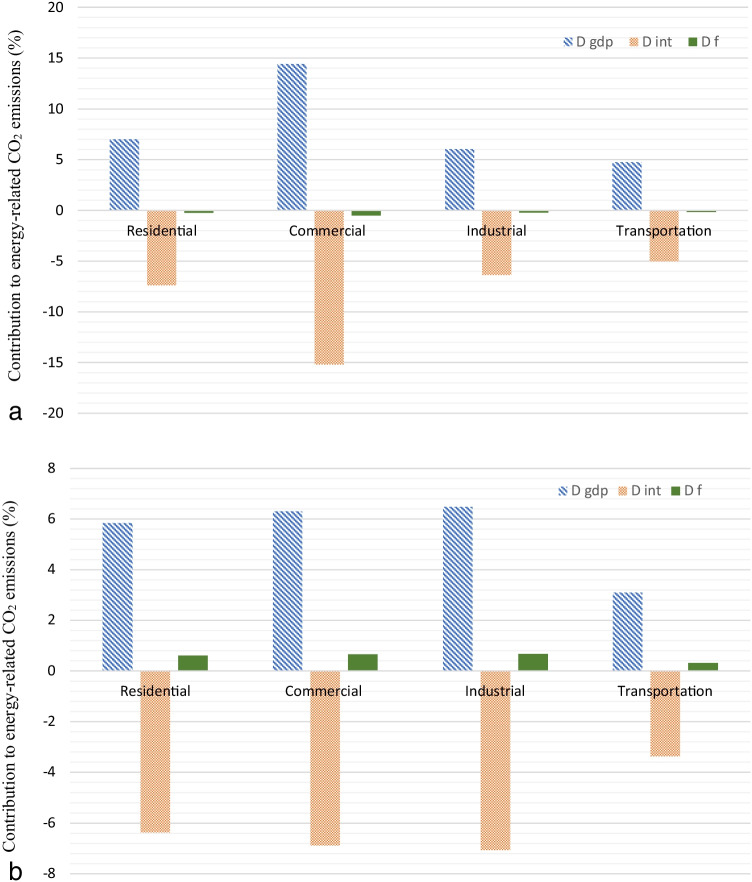
**a** Sectoral decomposition analysis on the final demand sectors in the USA, 2000–2017. **b** Sectoral decomposition analysis on the final demand sectors in Germany, 2000–2017

In this subsection, we present the results of the sectoral DA in the USA and Germany from 2000 to 2017 (Figures 6a and b).

From the sectoral analysis, the following remarks can be obtained:

- The GDP effect of commercial sector in the US economy is the major contributing factor on energy-related CO_2_ emissions (+ 14.41%). On the contrary, the energy intensity in the commercial sector has a considerable negative effect on energy-related CO_2_ emissions (− 15.19%). For the German economy, the GDP effect of industrial (6.48%) and commercial (6.31%) sectors has the most positive effect on energy-related CO_2_ emissions. Also, the energy intensity in the same sectors presents the major negative contribution (− 7.07% and 6.88%, respectively). Changes in emission factor have minor influence on energy-related CO_2_ emissions, in all sectors for both economies. The energy intensity effect contributes similarly in the mitigation of CO_2_ emissions in all sectors. Changes in the structure of industry and commercial sectors are crucial for future reductions in CO_2_ emissions, especially for the German economy. Regarding the above-mentioned facts, an important policy recommendation is the implementation of continuous improvements in reducing carbon intensity through fuel switching.
- Transportation sector has the minor contribution to the change of energy-related CO_2_ emissions in both economies.

### Comparison of the results with other studies

The findings of our analysis are in accordance with similar recent works; characteristically Sadorsky (2020) concludes that the key driver for the decrease of CO_2_ emissions was the declining energy intensity, while GDP was the biggest contributor on enlarging CO_2_ emissions. Wang et al. (2020), Wang et al. (2018), and Gonzalez et al. (2014) reported similar findings.

Regarding sectoral analysis, our results are similar with the work Wang et al. (2020) and Shahiduzzaman and Layton (2017) for the USA; energy intensity and GDP effects are presented as the main driving forces responsible for the decrease and increase of energy-related CO_2_ emissions, respectively, especially in industrial and commercial sectors. The sectoral analysis for the German economy gives similar results with Marrero and Ramos-Real (2013), Obadi and Korček (2015), and Trotta (2019), denoting that the growth of the commercial sector during the study period is not accompanied by corresponding measures to improve energy efficiency, leading in the increase in energy-related CO_2_ emissions in this sector.

Although there are various studies focusing on driving factors of energy-related CO_2_ emissions in comparison level for several major economies, a comparison exclusively for German and the US economies is not presented in the literature. The scientific interest for these two economies is well known; the different structures and dependencies on energy while they are among the world’s economic and geopolitical leading players.

## Conclusions

The US and German economies for the period 2000–2017 present an increasing GDP and income, with their population increased (15.2%) in the USA while remain stable in Germany. The decoupling analysis indicates a transition from strong to weak decoupling for both the USA and to persist in Germany during the period examined. This might be the result of the recession period where the income reduced more rapidly than CO_2_ emissions.

We employ LMDI additive and multiplicative techniques to identify the contributing factors on the evolution of energy-related CO_2_ emissions. The crucial factor responsible for the decrease of CO_2_ emissions for both economies is the declining energy intensity. The leading role has been the declining trends of the EI industrial sector which assumed to be the most energy intensive sector. The energy intensity of industrial sector is declined by 34% for the German economy and by 12% for the US economy. Remarkably, Germany, although increased the share of its industrial sector, reduced more intensively CO_2_ emissions, mainly due to intensive decreasing EI of the industrial sector. This performance of the industrial sector of Germany, combined with shifts of the energy mix, offsets the increased contribution of the income factor which had a much stronger influence compared to the US income. This made Germany to achieve a higher reduction in carbon intensity although its higher percentage income increases. As the transportation sector has the minor contribution to energy-related CO_2_ emission mitigation in both economies, corresponding measures have to be taken, e.g., the usage of cleaner forms of energy (fuel switching), the adoption of efficient technology.

As economic, environmental, and human systems are strongly coupled, the analysis should take into account the population trends. The impact of the population has been strong in the USA, however, negligible in Germany mainly because of the population in Germany remains almost stable while there is a substantially increasing population in the USA; there evolutions provide evidence that CO_2_ emissions are the outcome of processes and interactions within coupled systems. Natural, economic, and social systems are coupled and interdependent. The adoption of an integrated approach in the design of energy policy may enrich potentials to reach the newly set ambitious climate targets which seems hard to achieve once past trends prevail, indicated by the “business as usual” scenarios of the present study. Therefore, nationally determined contributions (NDCs) are considered to be revised for both economies, focused on realistic and achievable environmental strategies. Investments in sustainable technologies could mitigate energy-related CO_2_ emissions, promoting low-carbon technology innovation and development.

The future research effort should include complementary research on the causal relationship between the driving forces of CO_2_ emissions using an econometric analysis. This could provide a holistic approach for a sustainable future, denoting which variables are highly determined by others and leading to specific policy measures.

### Supplementary Information

ESM 1 (PDF 268 kb)

## Data Availability

The raw data used in this study are available online at the relevant reference links. The data that support the findings of this study are available from the corresponding author upon reasonable request. Supporting Material may be found online in the Supporting Information section at the end of this article.

## Appendix 1

In multiplicative LMDI technique, the change scheme is

22 $${D}_{tot}={D}_p{D}_{inc}{D}_{int}{D}_f{D}_s={C}_T/{C}_0$$

where *D*_tot_ is the change of total CO_2._

The relevant formulas for the decomposition factors are as follows:

23 $${D}_p=\exp \left({\sum}_{i=1}^4\frac{\left({C}_{i,T}-{C}_{i,0}\right)/\left(\ln {C}_{i,T}-\ln {C}_{i,0}\right)}{\left({C}_T-{C}_0\right)/\left(\ln {C}_T-\ln {C}_0\right)}\ln \left(\frac{P_T}{P_0}\right)\right)$$

24 $${D}_{\mathrm{inc}}=\exp \left({\sum}_{i=1}^4\frac{\left({C}_{i,T}-{C}_{i,0}\right)/\left(\ln {C}_{i,T}-\ln {C}_{i,0}\right)}{\left({C}_T-{C}_0\right)/\left(\ln {C}_T-\ln {C}_0\right)}\ln \left(\frac{\mathrm{In}{c}_T}{\mathrm{In}{c}_0}\right)\right)$$

25 $${D}_{\mathrm{int}}=\exp \left({\sum}_{i=1}^4\frac{\left({C}_{i,T}-{C}_{i,0}\right)/\left(\ln {C}_{i,T}-\ln {C}_{i,0}\right)}{\left({C}_T-{C}_0\right)/\left(\ln {C}_T-\ln {C}_0\right)}\ln \left(\frac{I_T}{I_0}\right)\right)$$

26 $${D}_{\mathrm{f}}=\exp \left({\sum}_{i=1}^4\frac{\left({C}_{i,T}-{C}_{i,0}\right)/\left(\ln {C}_{i,T}-\ln {C}_{i,0}\right)}{\left({C}_T-{C}_0\right)/\left(\ln {C}_T-\ln {C}_0\right)}\ln \left(\frac{F_{i,T}}{F_{i,0}}\right)\right)$$

27 $${D}_{\mathrm{s}}=\exp \left({\sum}_{i=1}^4\frac{\left({C}_{i,T}-{C}_{i,0}\right)/\left(\ln {C}_{i,T}-\ln {C}_{i,0}\right)}{\left({C}_T-{C}_0\right)/\left(\ln {C}_T-\ln {C}_0\right)}\ln \left(\frac{S_{i,T}}{S_{i,0}}\right)\right)$$

where *D*_p_ is the change of population, *D*_inc_ the change of income, *D*_int_ the change of the energy intensity, *D*_f_ the change of the emission factor, and *D*_s_ the change of the energy structure.

## Footnotes

1 https://www4.unfccc.int/sites/submissions/indc/Submission%20Pages/submissions.aspx

2 https://data.worldbank.org

## References

[CR1] Ang BW, van den Bergh JCJM (1999). Decomposition methodology in energy demand and environmental analysis. Handbook of environmental and resource economics.

[CR2] Bachtrögler J (2016). On the effectiveness of EU structural funds during the Great Recession: Estimates from a heterogeneous local average treatment effects framework. WU Vienna University of Economics and Business.

[CR3] Bailey I, Rupp S (2004). Politics, industry and the regulation of industrial greenhouse-gas emissions in UK and Germany. Eur Environ.

[CR4] Baldwin JG, Wing IS (2013). The spatiotemporal evolution of U.S. carbon dioxide emissions: stylized facts and implications for climate policy. J Reg Sci.

[CR5] Bhattacharyya SC, Matsumura W (2010). Changes in the GHG emission intensity in EU-15: lessons from a decomposition analysis. Energy.

[CR6] Bithas K, Kalimeris P (2013). Re-estimating the decoupling effect: is there an actual transition towards a less energy-intensive economy?. Energy.

[CR7] Bithas K, Kalimeris P (2018). Unmasking decoupling: Redefining the Resource Intensity of the Economy. Sci Total Environ.

[CR8] Blodgett J, Parker L, and Industry Division (2002) Global climate change: U.S. greenhouse gas emissions —status, trends, and projections. CRS Report for Congress, Resources, Science, and Industry Division. https://crsreports.congress.gov

[CR9] Bureau of Economic Analysis (BEA) (2020) BEA Data. https://www.bea.gov/data/gdp. Accessed 3 Aug 2021

[CR10] Cohen G, Jalles JT, Loungani P, Marto R (2018). The long-run decoupling of emissions and output: evidence from the largest emitters. Energy Policy.

[CR11] European Commission (2020a) European climate law – achieving climate neutrality by 2050. Law, Published Initiatives. https://ec.europa.eu/info/law/better-regulation/have-your-say/initiatives/12108-European-climate-law-achieving-climate-neutrality-by-2050. Accessed 3 Aug 2021

[CR12] European Commission (2020b) Eurostat Database. https://ec.europa.eu/eurostat/data/database. Accessed 5 May 2021

[CR13] Fatima T, Xia E, Cao Z, Khan D, Fan JL (2019). Decomposition analysis of energy-related CO_2_ emission in the industrial sector of China: evidence from the LMDI approach. Environ Sci Pollut Res.

[CR14] Federal Ministry for Economic Affairs and Energy (BMWI) (2019) A modern industrial policy. Industrial Policy. https://www.bmwi.de/Redaktion/EN/Dossier/modern-industry-policy.html. Accessed 3 Aug 2021

[CR15] Federal Ministry for the Environment, Nature Conservation, Building and Nuclear Safety (BMUB) (2016) Climate Action Plan 2050, Principles and goals of the German government’s climate policy. Public Relations Division. https://www.bmuv.de/fileadmin/Daten_BMU/Pools/Broschueren/klimaschutzplan_2050_en_bf.pdf. Accessed 5 May 2021

[CR16] Gonzalez PF, Landajo M, Presno MJ (2014). The driving forces behind changes in CO_2_ emission levels in EU-27. Differences between member states. Environ Sci Policy.

[CR17] Haein K, Minsang K, Hyunggeun K, Sangkyu P (2020). Decomposition analysis of CO_2_ emission from electricity generation: comparison of OECD countries before and after the financial crisis. Energies.

[CR18] Hatzigeorgiou E, Polatidis H, Haralambopoulos D (2008). CO_2_ emissions in Greece for 1990–2002: a decomposition analysis and comparison of results using the arithmetic mean divisia index and logarithmic mean divisia index techniques. Energy.

[CR19] Hatzigeorgiou E, Polatidis H, Haralambopoulos D (2010). Energy CO_2_ emissions for 1990–2020: a decomposition analysis for EU-25 and Greece. Energy Sources A: Recov Util Environ Eff.

[CR20] Herrendorf B, Rogerson R, Valentinyi A, Aghion P, Durlauf SN (2014). Growth and structural transformation. Handbook of economic growth.

[CR21] Hu P, Zhou Y, Gao Y, Zhou J, Wang G, Zhu G (2021) Decomposition analysis of industrial pollutant emissions in cities of Jiangsu based on the LMDI method. Environ Sci Pollut Res. 10.1007/s11356-021-15741-1 10.1007/s11356-021-15741-1 34370201

[CR22] Intergovernmental Panel on Climate Change (IPCC) (2006). IPCC guidelines for national greenhouse gas inventories.

[CR23] International Energy Agency (IEA) (2020a) Data & Statistics. https://www.iea.org/data-and-statistics. Accessed 5 May 2021

[CR24] International Energy Agency (IEA) (2020b) Germany 2020 Energy Policy Review. https://www.iea.org/reports/germany-2020

[CR25] Jiang R, Li R, Wu Q (2019). Investigation for the decomposition of carbon emissions in the USA with C-D function and LMDI methods. Sustainability.

[CR26] Lu Y, Khan Z, Alvarez-Alvarado M, Zhang Y, Huang Z, Imran MA (2020). Critical review of sustainable energy policies for the promotion of renewable energy sources. Sustainability.

[CR27] Marrero GA, Ramos-Real FJ (2013). Activity sectors and energy intensity: decomposition analysis and policy implications for European countries (1991–2005). Energies.

[CR28] Martínez DM, Ebenhack BW, Wagner TP (2019). Chapter 1 - Introductory concepts, Energy Efficiency.

[CR29] Obadi SM, Korček M (2015) Investigation of driving forces of energy consumption in EU 28 countries. Int J Energy Econ Policy 5(2):422–432. https://www.econjournals.com/index.php/ijeep/article/view/1086. Accessed 1 Aug 2022

[CR30] Sadorsky P (2020). Energy related CO_2_ emissions before and after the financial crisis. Sustainability.

[CR31] Shahiduzzaman M, Layton A (2017). Decomposition analysis for assessing the United States 2025 emissions target: how big is the challenge?. Renew Sust Energ Rev.

[CR32] Song Y, Zhang M, Zhou M (2019). Study on the decoupling relationship between CO_2_ emissions and economic development based on two-dimensional decoupling theory: A case between China and the United States. Ecol Indic.

[CR33] Tapio P (2005). Towards a theory of decoupling: degrees of decoupling in the EU and the case of road traffic in Finland between 1970 and 2001. Transp Policy.

[CR34] Telli A, Erat S, Demir B (2020). Comparison of energy transition of Turkey and Germany: energy policy, strengths/weaknesses and targets. Clean Techn Environ Policy.

[CR35] The World Bank Group (2020) World bank open data. https://data.worldbank.org. Accessed 15 Apr 2021

[CR36] Tol RSJ, Pacala SW, Socolow RH (2009). Understanding long-term energy use and carbon dioxide emissions in the USA. J Policy Model.

[CR37] Trotta G (2019). Assessing energy efficiency improvements, energy dependence, and CO2 emissions in the European Union using a decomposition method. Energy Eff.

[CR38] U.S Energy Information Administration (2020a) Use of energy explained. International Statistics & Analysis. https://www.eia.gov/energyexplained/use-of-energy/. Accessed 15 Apr 2021

[CR39] U.S Energy Information Administration (2020b) U.S. energy-related carbon dioxide emissions, 2019. Analysis & Projections. https://www.eia.gov/environment/emissions/carbon/. Accessed 15 April 2021

[CR40] van Neuss L (2019). The drivers of structural change. J Econ Surv.

[CR41] Vinuya F, DiFurio F, Sandoval E (2010). A decomposition analysis of CO_2_ emissions in the United States. Appl Econ Lett.

[CR42] Wang C, Chen J, Zou J (2005). Decomposition of energy-related CO_2_ emission in China: 1957–2000. Energy.

[CR43] Wang Q, Zhao M, Li R, Su M (2018). Decomposition and decoupling analysis of carbon emissions from economic growth: a comparative study of China and the United States. J Clean Prod.

[CR44] Wang Z, Jiang Q, Dong K, Mubarik MS, Dong X (2020). Decomposition of the US CO_2_ emissions and its mitigation potential: an aggregate and sectoral analysis. Energy Policy.

[CR45] Ward JD, Sutton PC, Werner AD, Costanza R, Mohr SH, Simmons CT (2016). Is decoupling GDP growth from environmental impact possible?. PLoS One.

[CR46] Watson MW (2014). Inflation persistence, the NAIRU, and the Great Recession. Am Econ Rev.

[CR47] Wu L, Kaneko S, Matsuoka S (2005). Driving forces behind the stagnancy of China’s energy-related CO_2_ emissions from 1996 to 1999: the relative importance of structural change, intensity change and scale change. Energy Policy.

[CR48] Yang P, Liang X, Drohan PJ (2020). Using Kaya and LMDI models to analyze carbon emissions from the energy consumption in China. Environ Sci Pollut Res.

[CR49] Yao C, Feng K, Hubacek K (2015). Driving forces of CO_2_ emissions in the G20 countries: an index decomposition analysis from 1971 to 2010. Ecol Inform.

[CR50] Yasmeen H, Wang Y, Zameer H, Solangi YA (2020). Decomposing factors affecting CO_2_ emissions in Pakistan: insights from LMDI decomposition approach. Environ Sci Pollut Res.

[CR51] Zhang J, Fan Z, Chen Y, Gao J, Liu W (2020). Decomposition and decoupling analysis of carbon dioxide emissions from economic growth in the context of China and the ASEAN countries. Sci Total Environ.

