# Supplementary material for: Carbon and energy intensity of the USA and Germany. A LMDI decomposition approach and decoupling analysis
Source: Environ Sci Pollut Res Int. 2022 Sep 15;30(5):12412–27. doi: 10.1007/s11356-022-22978-x (PMC9898335; doi:10.1007/s11356-022-22978-x)
Supplement: Supplementary file 1 — (PDF 268 kb) [file 11356_2022_22978_MOESM1_ESM.pdf]

Carbon and Energy Intensity of the USA and Germany. A LMDI decomposition approach and decoupling analysis.

Eleni Koilakou[a*], Emmanouil Hatzigeorgiou[a,b], Kostas Bithas[a]

a. Institute of Urban Environment & Human Resources, Department of Economic & Regional Development, Panteion University, 29 Aristotelous Street, GR-17671, Kallithea, Athens, Greece

b. Energy Management Laboratory, Department of Environment, University of the Aegean, University Hill, 81100, Lesvos, Greece

*Corresponding author at: Institute of Urban Environment & Human Resources, Department of Economic & Regional Development, Panteion University, 29 Aristotelous Street, GR-17671, Kallithea, Athens, Greece.
E-mail addres: elenikoilakou@gmail.com .

Supporting Information File

All the indexed ratios in figures are calculated to a base year (base year = t_0=100):

$$\text{Indexed Value } t_1 = 100 + \frac{(Value_{t1} - Value_{t0})}{Value_{t0}}$$

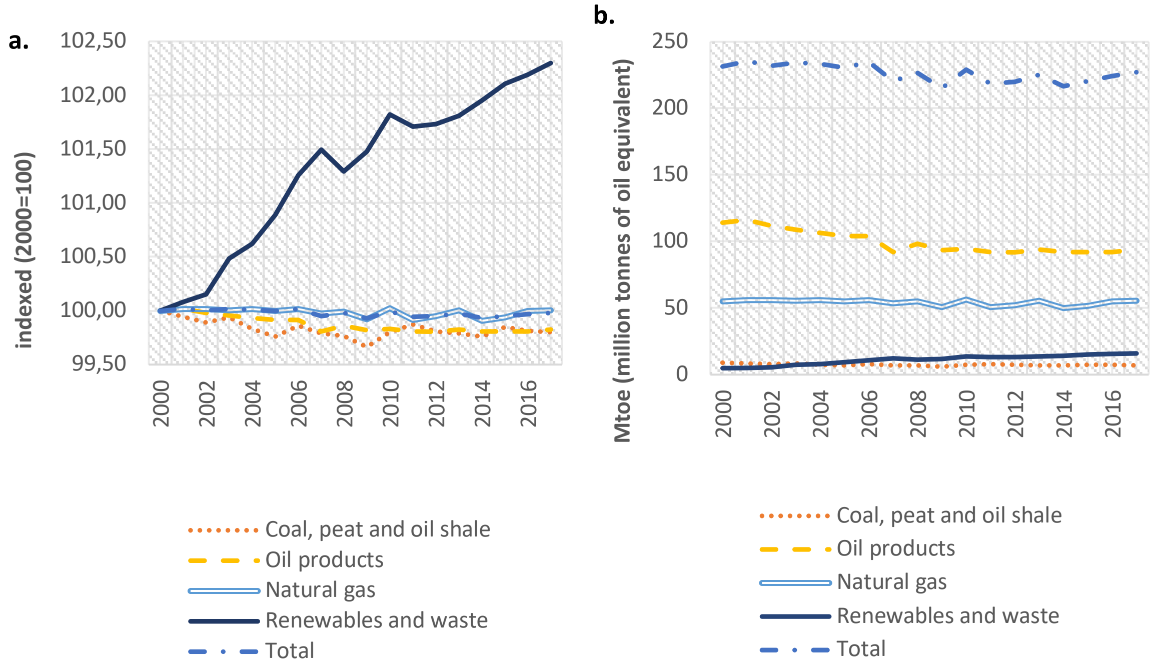

Fig.S1. Energy consumption per fuel in Germany from 2000 to 2017.

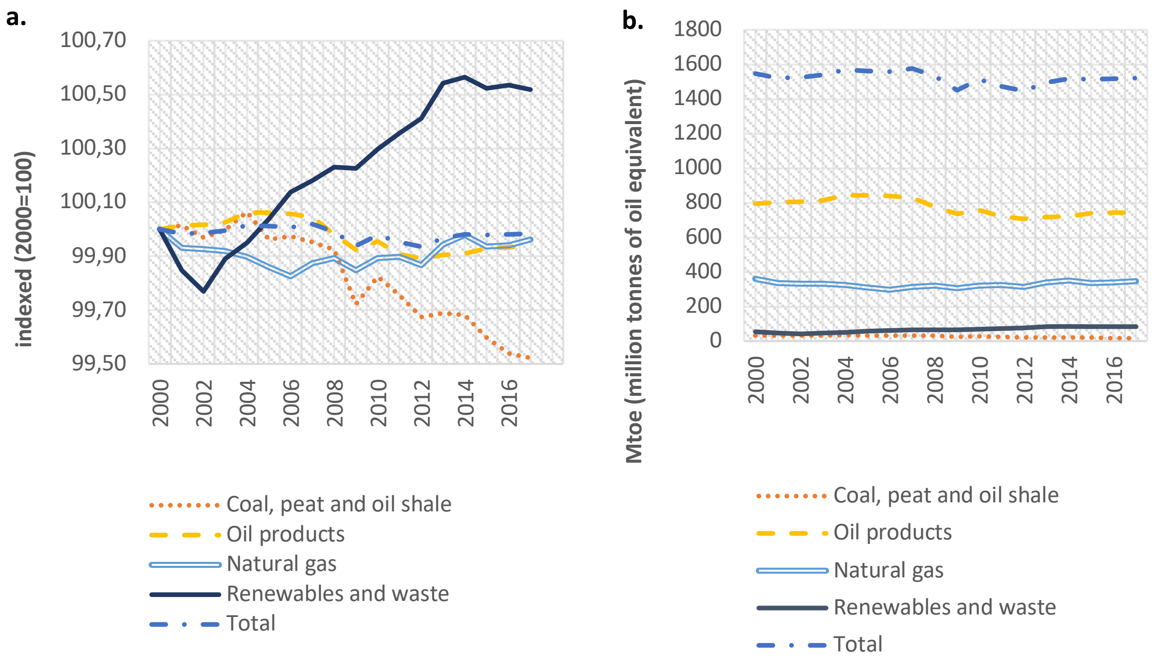

Fig.S2. Energy consumption per fuel in the USA from 2000 to 2017.

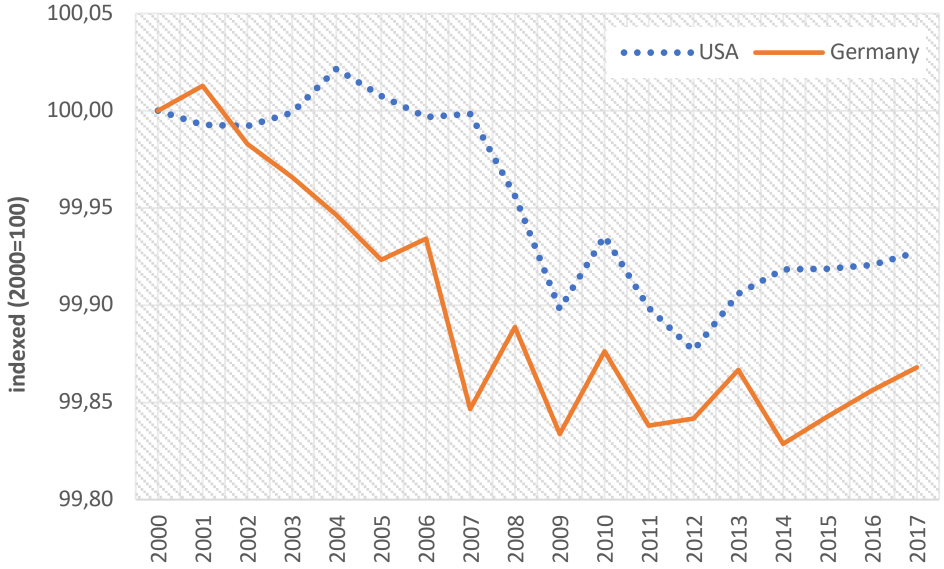

Fig.S3. Energy-related CO_2 emissions in the USA and Germany from 2000 to 2017 (indexed 2000=100).

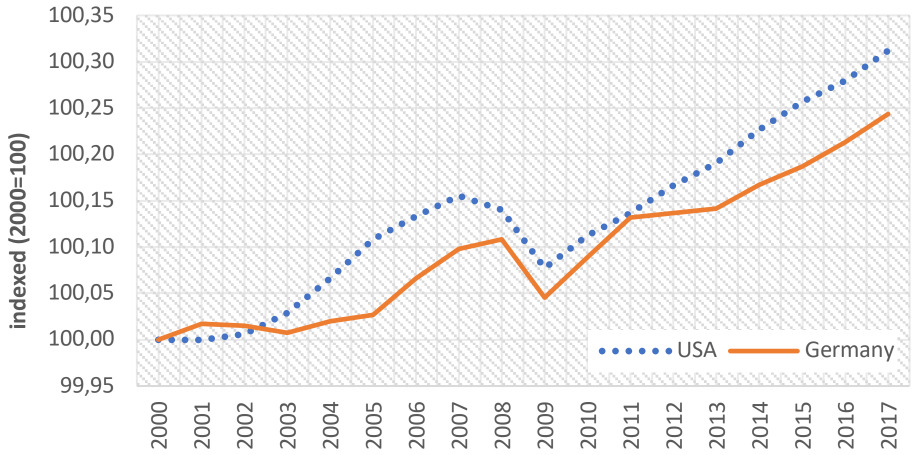

Fig.S4. GDP in the USA and Germany from 2000 to 2017 (indexed 2000=100).

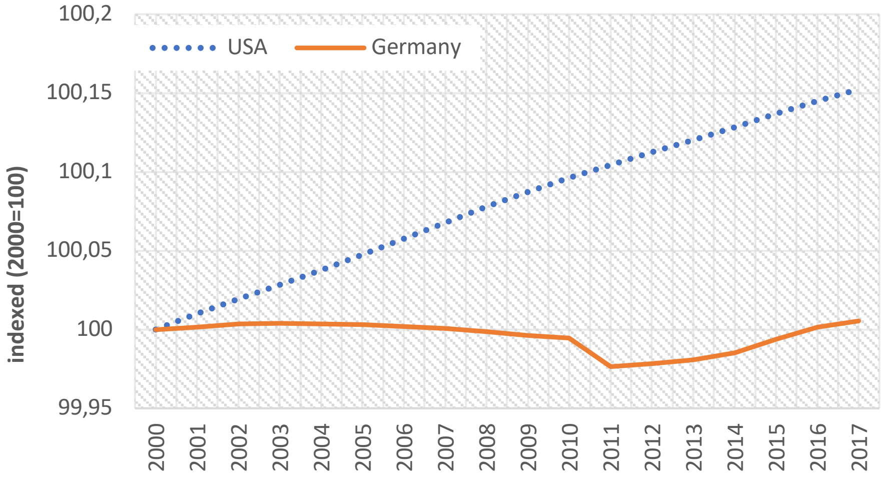

Fig.S5. Population in the USA and Germany from 2000 to 2017 (indexed 2000=100).

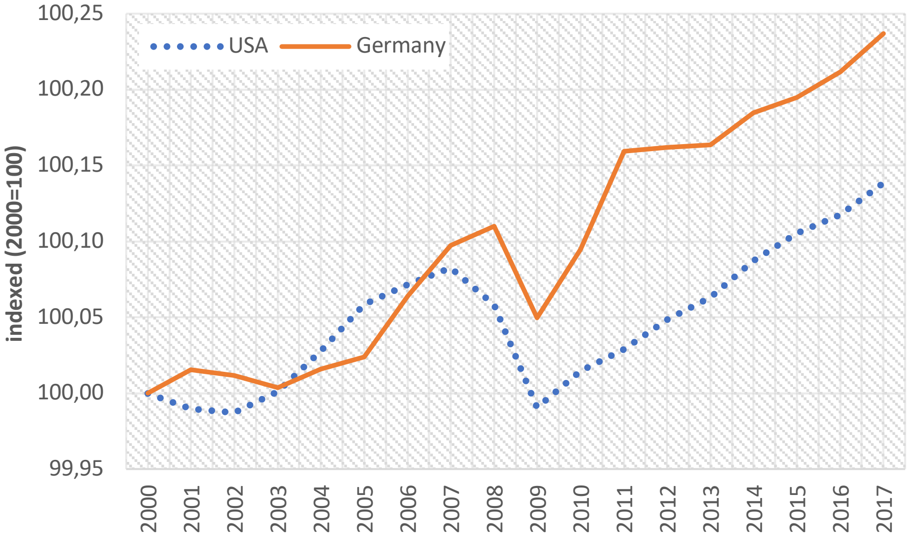

Fig.S6. Income in the USA and Germany from 2000 to 2017 (indexed 2000=100).

Fuel	Coefficient
Coal	3.99 tCO_2/toe
Oil	3.07 tCO_2/toe
Natural Gas	2.35 tCO_2/toe
Renewable Resources	0.00 tCO_2/toe

Table S1. CO2 emission coefficients

References

Bureau of Economic Analysis (BEA) (2020) BEA Data. https://www.bea.gov/data/gdp

Intergovernmental Panel on Climate Change (IPCC) (2006) IPCC guidelines for national greenhouse gas inventories. Intergovernmental panel on climate change. London.

International Energy Agency (IEA) (2020a) Data & Statistics. https://www.iea.org/data-and-statistics

The World Bank Group (2020) World Bank Open Data. https://data.worldbank.org
